# Supplementary material for: Realizing reversible phase transformation of shape memory ceramics constrained in aluminum
Source: Nat Commun. 2023 Nov 4;14:7103. doi: 10.1038/s41467-023-42815-0 (PMC10625574; doi:10.1038/s41467-023-42815-0)
Supplement: Supplementary file 1 — Supplementary Information [file 41467_2023_42815_MOESM1_ESM.pdf]

Supplementary Information

Realizing reversible phase transformation of shape memory ceramics constrained in aluminum

Wangshu Zheng[1], Yan Shi[1,2], Lei Zhao[1], Shuangyue Jia[1], Linghai Li[1], Chee Lip Gan[3*], Di Zhang[1*], Qiang Guo[1*]

[1]State Key Lab of Metal Matrix Composites, Shanghai Jiao Tong University, 800 Dongchuan Road, Shanghai, 200240, China

[2]Zhejiang Academy of Special Equipment Science, 211 Kaixuan Road, Hangzhou, 310020, China

[3]School of Materials Science and Engineering, Nanyang Technological University, 639798, Singapore, Singapore

*Corresponding authors: clgan@ntu.edu.sg (C. L. Gan); zhangdi@sjtu.edu.cn (D. Zhang); guoq@sjtu.edu.cn (Q. Guo).

This file includes:

Supplementary Methods

Supplementary Figs. 1 to 15

Supplementary Tables 1 to 3

Supplementary Methods

Calculation of dislocation density in the CZ/Al composites

The full-width at half-maximum (FWHM) of the peaks is obtained by deducting the instrumental broadening. Grain refinement and dislocations multiplication or annihilation are the main reasons leading to the broadening of aluminum (Al) peaks in the composites. According to the Williamson-Hall method[1], the dislocation density can be estimated from peak broadening in the X-ray Diffraction (XRD) pattern:

$$B\cos\theta = 2\varepsilon\sin\theta + \frac{\lambda}{d} \tag{1}$$

$$\rho = \frac{\sqrt{3}\varepsilon}{db\sin\theta} \tag{2}$$

where is ρ is the dislocation density, b= 2.86 × 10^{-10} m is the Burger's vector magnitude, d is the average grain size, B is the FWHM of diffraction peak, λ is the wavelength of Cu Kα radiation (1.54 Å), and θ is the Bragg angle.

Estimation of Young's modulus of the CZ/Al micro-pillars

The Young's modulus was estimated from true compressive stress-strain (σ-ε) curves of CZ/Al micro-pillars. In detail, the unloading curve was magnified in Supplementary Fig. 9. Young's modulus E was deducted by the tangent method from the unloading segment of the pillars:

$$E = \frac{\partial \sigma}{\partial \varepsilon} \tag{3}$$

In spite that Young's modulus of the micro-pillars is lower than that of the bulk sample via nanoindentation test, one could still infer that the micro-pillar which has higher Young's modulus possessed higher cerium-doped shape memory zirconia (CZ) content.

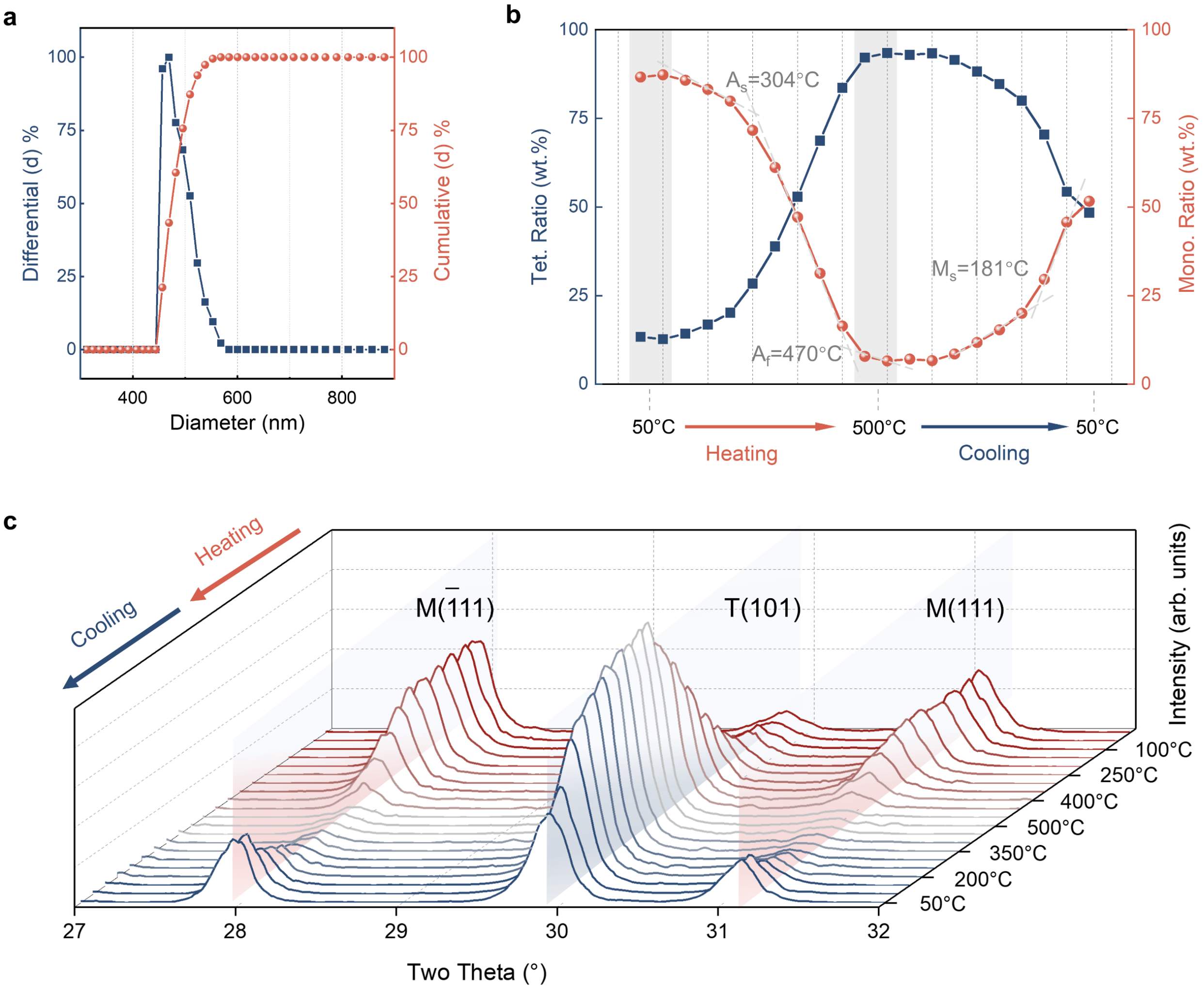

Supplementary Fig. 1 | Diameter distribution, phase composition and thermally induced martensitic transformation of cerium-doped shape memory zirconia (CZ). a Diameter distribution of CZ via particle size analyzer (Omni, Brookhaven), where the average particle size of CZ was measured to be approximately 480 nm. **b** Phase composition of CZ extracted from the monoclinic (M) and tetragonal (T) characteristic peak in **c** in-situ high-temperature XRD spectrums. The austenite start and finish temperatures (A_s and A_f) and the martensite start temperature (M_s) of the CZ particles were measured to be A_s = 304°C, A_f = 470°C, and M_s = 181°C, respectively, while the martensite finish temperature (M_f) was lower than room temperature and cannot be obtained.

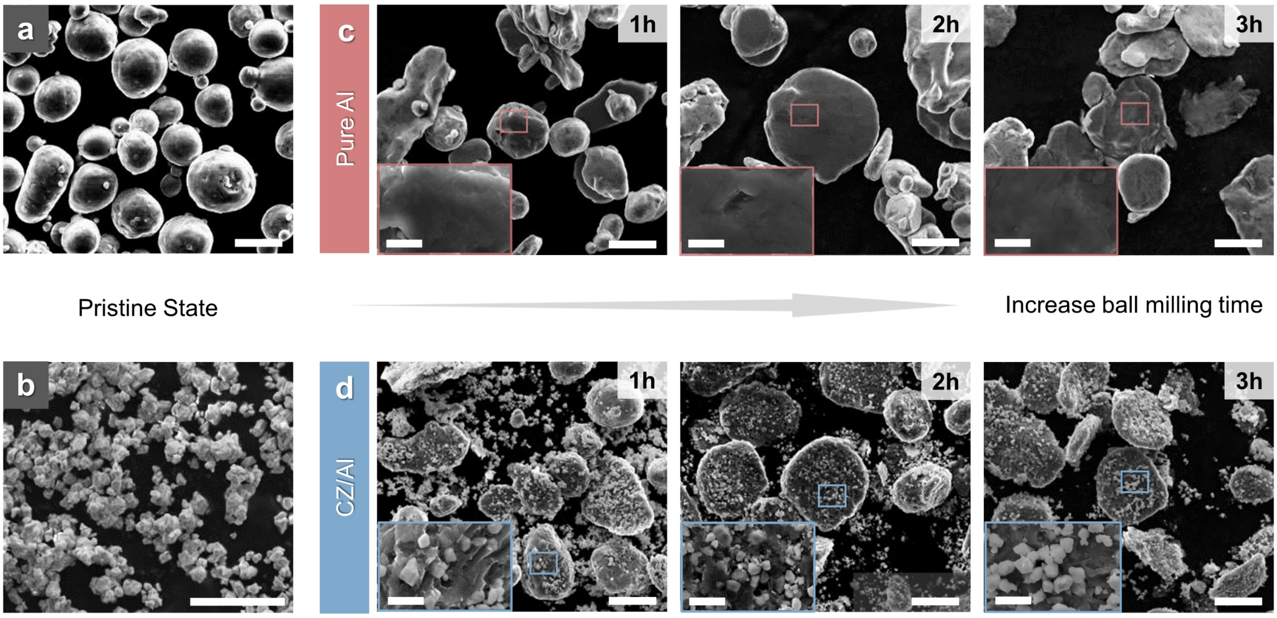

Supplementary Fig. 2 | Evolution on the morphology of the composite powders at pristine state and during ball milling. Scanning electron microscopy (SEM) image of **a** pristine Al powders, **b** pristine CZ particles and **c** pure Al powders, **d** CZ/Al composite powders with the milling times of 1 h, 2 h, 3 h. After ball milling for 3 hr, the Al powders were reshaped into thin flakes, and the CZ particle clusters were embedded on the surface of these Al flakes. The scale bars of **a**, **b**, **c** and **d**, **c** and **d** insets are 10, 5, 10, 2 μm, respectively.

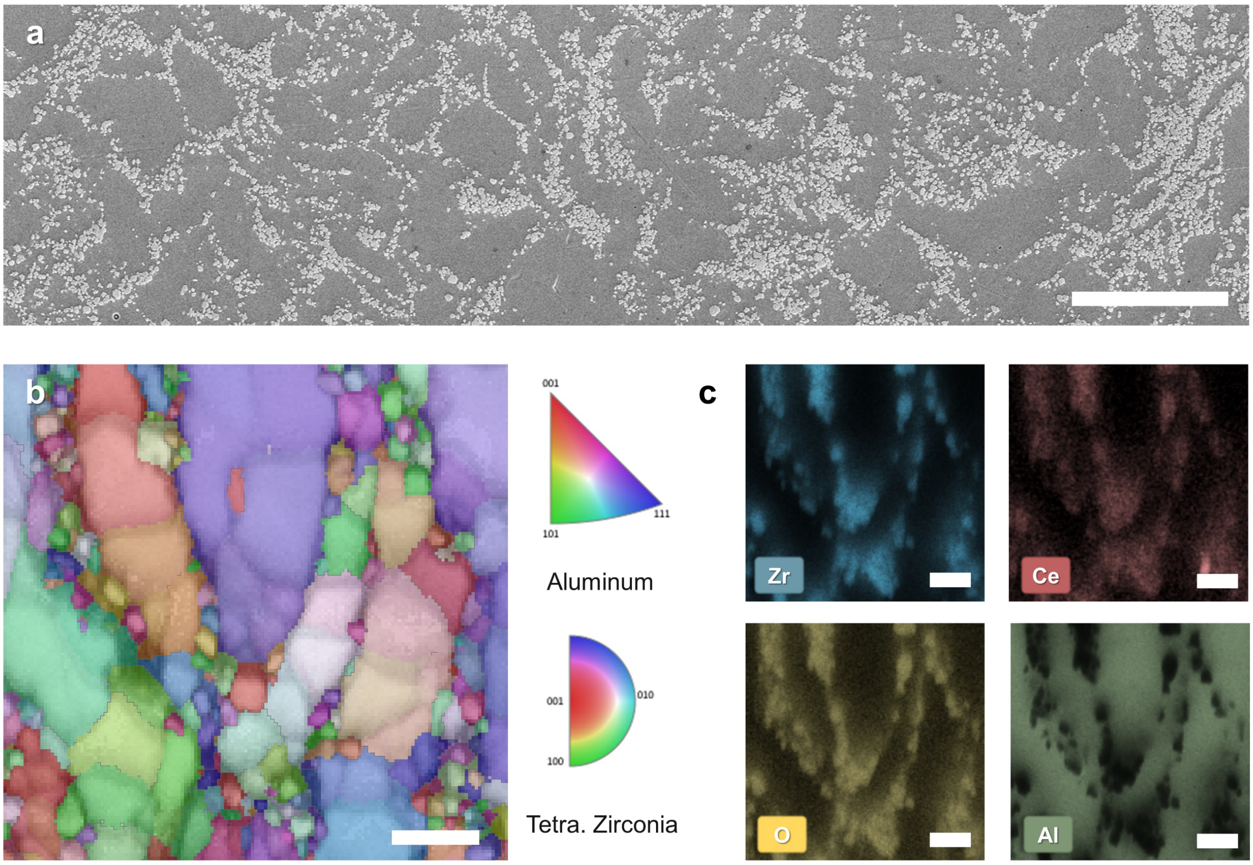

Supplementary Fig. 3 | Morphology, crystal orientation, and element distribution of as fabricated CZ/Al composites at grain scale. a SEM image of CZ/Al with a large view, showing the typical particle network structure at two-dimensional (2D) plane. **b** Electron back scattering diffraction (EBSD) of CZ/Al in a magnified region with **c** corresponding elemental mapping by energy dispersive spectroscopy (EDS) in SEM, indicating that the CZ particles were preferably dispersed along the Al grain boundary. The scale bars of **a**, **b**, **c** are 20, 2, 2 μm, respectively.

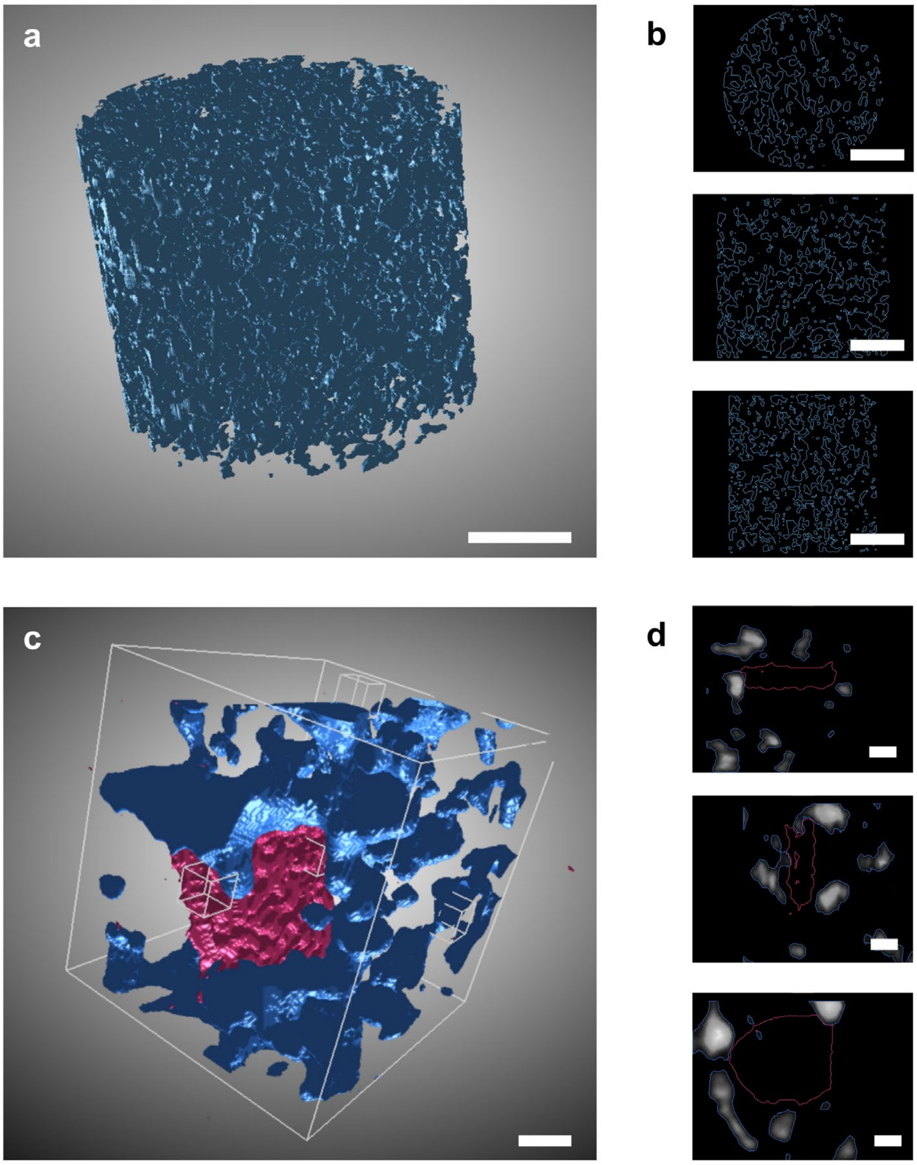

Supplementary Fig. 4 | Three-dimensional (3D) microstructure of as-fabricated CZ/Al composites. **a** X-ray computed tomography microscopy (XRT) volume renderings of CZ/Al bulk sample, with CZ particles meshed (blue) specifically and **b** corresponding 2D projections in the XY, XZ, YZ planes. **c** The representative magnified area with aluminum grains (red) surrounded by CZ particles and **d** corresponding 2D projections. The CZ particles in front of the Al grains were purposely removed to ensure a clearer view. The volume fraction of CZ in the composite was determined to be ~15.4% by XRT, aligning well with the prescribed value from composite fabrication (~16.7%). The scale bars of **a** and **b**, **c** and **d** are 100, 10 μm, respectively.

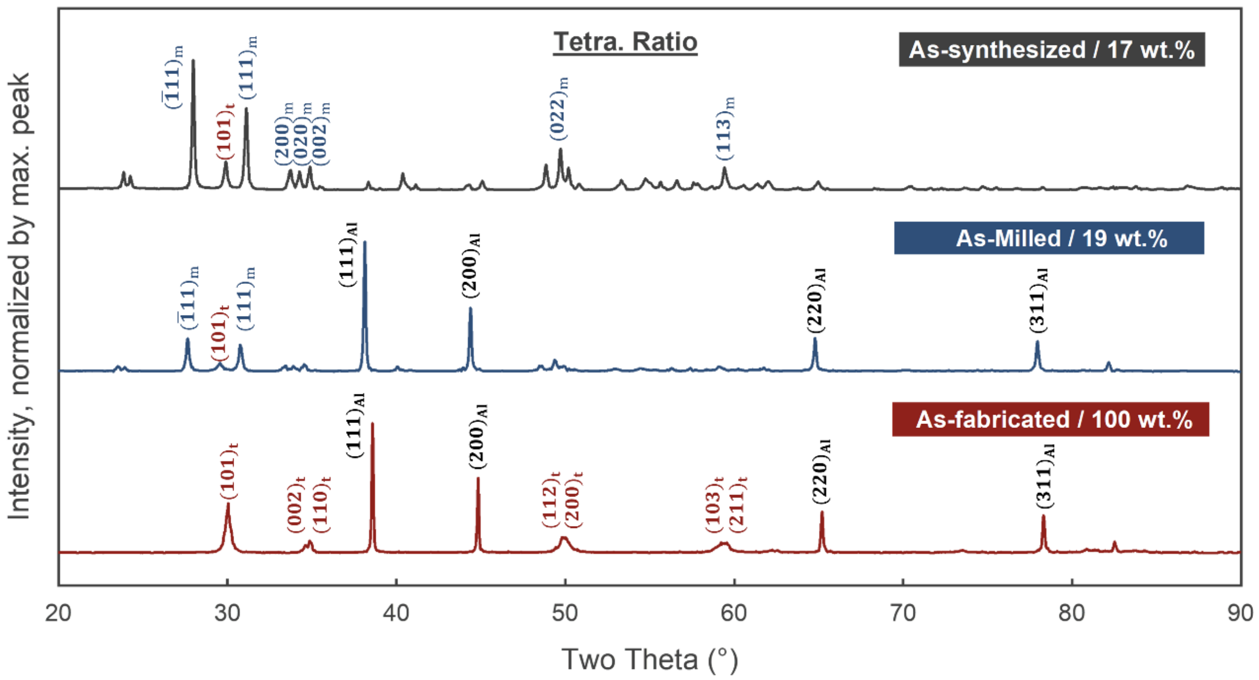

Supplementary Fig. 5 | Evolution on the phase constitute of the shape memory zirconia during fabrication. XRD spectrums of as-synthesized shape memory zirconia, as-milled composite powders, and as-fabricated composite. Compared to the pristine state, the CZ particles in the as-milled CZ/Al composite powders had a slight increase in the fraction of the tetragonal (T) phase to 19 wt.%. Upon hot pressing and subsequent cooling to room temperature, all the monoclinic (M) phases in the CZ/Al composite were found to transform to the tetragonal phase. No Al_3Zr characteristic peak was observed in the patterns, indicating that the content of this interfacial reaction product was lower than the detection limit (~1 wt.%, if any). Al – aluminum.

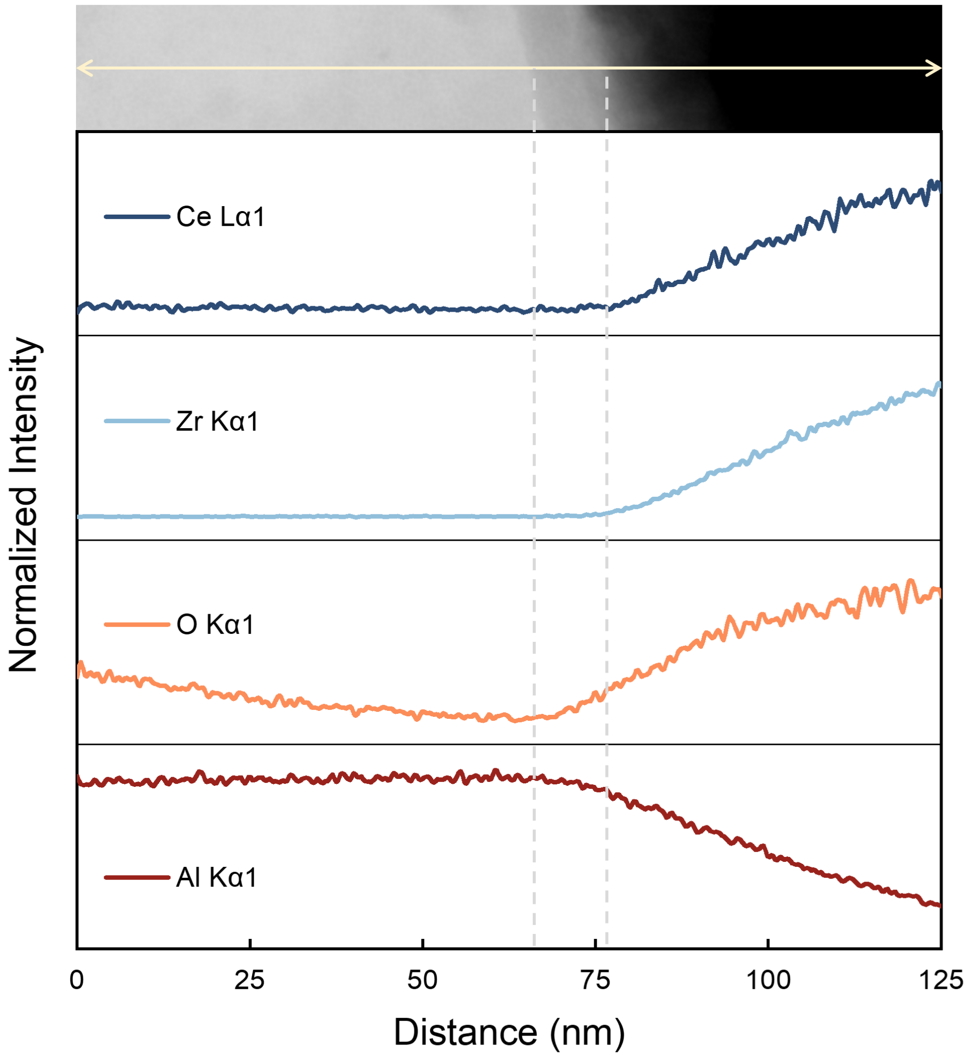

Supplementary Fig. 6 | Characterization on the interfacial layer in the as-fabricated CZ/Al composites. Scanning transmission electron microscopy (STEM)-EDS linear mapping on CZ/Al composites, showing the normalized intensity of Ce, Zr, O, Al elements with the distance, as evidence of the Al_2O_3 interfacial layer with ~10 nm thickness, which may come from the oxidization of Al and may enhance the cohesion between the CZ particles and the Al matrix.

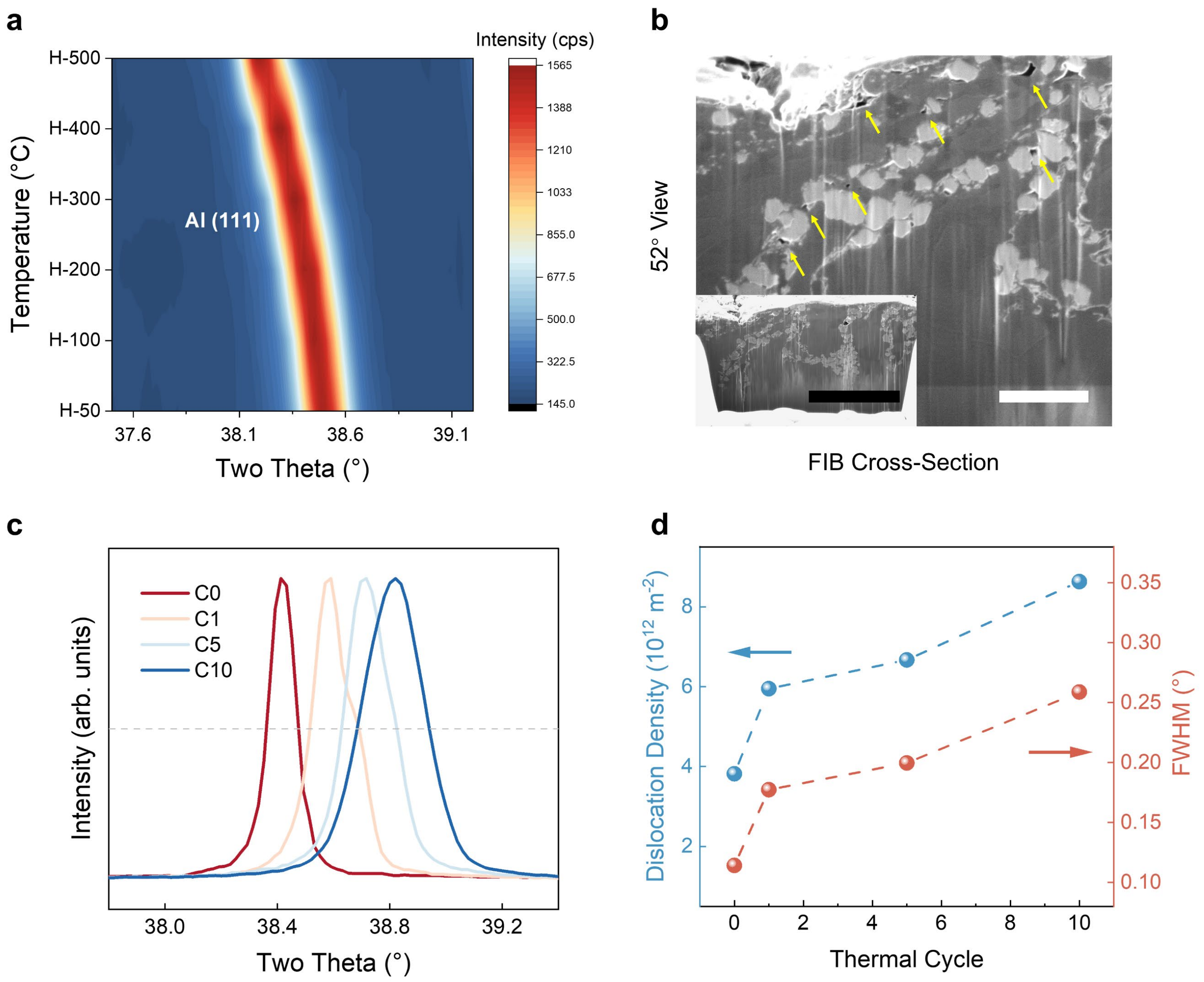

Supplementary Fig. 7 | Thermal response and post-mortem characterization on the bulk composites. a Contour map of 2θ-temperature-intensity obtained from *in-situ* high-temperature XRD measurement of Al (111) peak. **b** Focus ion beam **(**FIB) image of the cross-section from CZ/Al composites after the 15th thermal cycle. The interfacial detachment or cracking was marked by yellow arrows. **c** Normalized Al (111) peaks under multiple thermal cycles in XRD spectrums. **d** Evolution of the dislocation density and the FWHM after the thermal cycles. The scale bars of **b**, **b** inset image are 2, 10 μm, respectively.

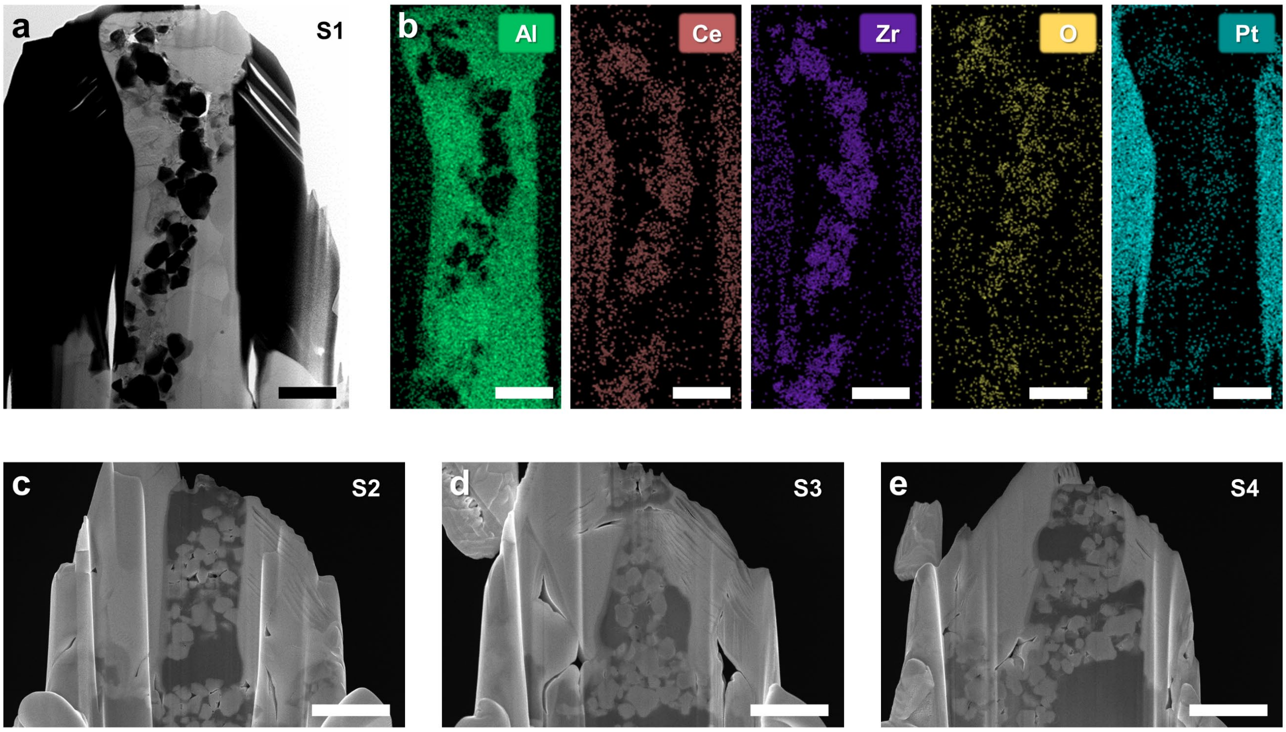

Supplementary Fig. 8 | Microstructure and element distribution in TEM cross-section of post-compression micro-pillar S1-S4. a Bright field (BF) image in STEM of post-compression micro-pillar S1 with **b** EDS mapping. SEM images on the cross-sections of post-compression micro-pillar **c** S2, **d** S3, and **e** S4 prepared via the lift-out method. The actual CZ volume fraction in pillars S1-S4 was approximately ~30 vol.%, 67 vol.%, 70 vol.%, and 55 vol.%, respectively, estimated by the area fractions of CZ and Al in the TEM cross-section. In spite of the different particle assembly patterns in the 3 pillars, most of the CZ particles were interconnected with each other, forming clusters embedded in the Al matrix (force-chain configuration[2]). In stark contrast from the geometries of pillar S1-S3, three unconnected CZ particle clusters were laminated with Al grains across the gauge length of pillar S4. The scale bars in **a-e** are all 1 μm.

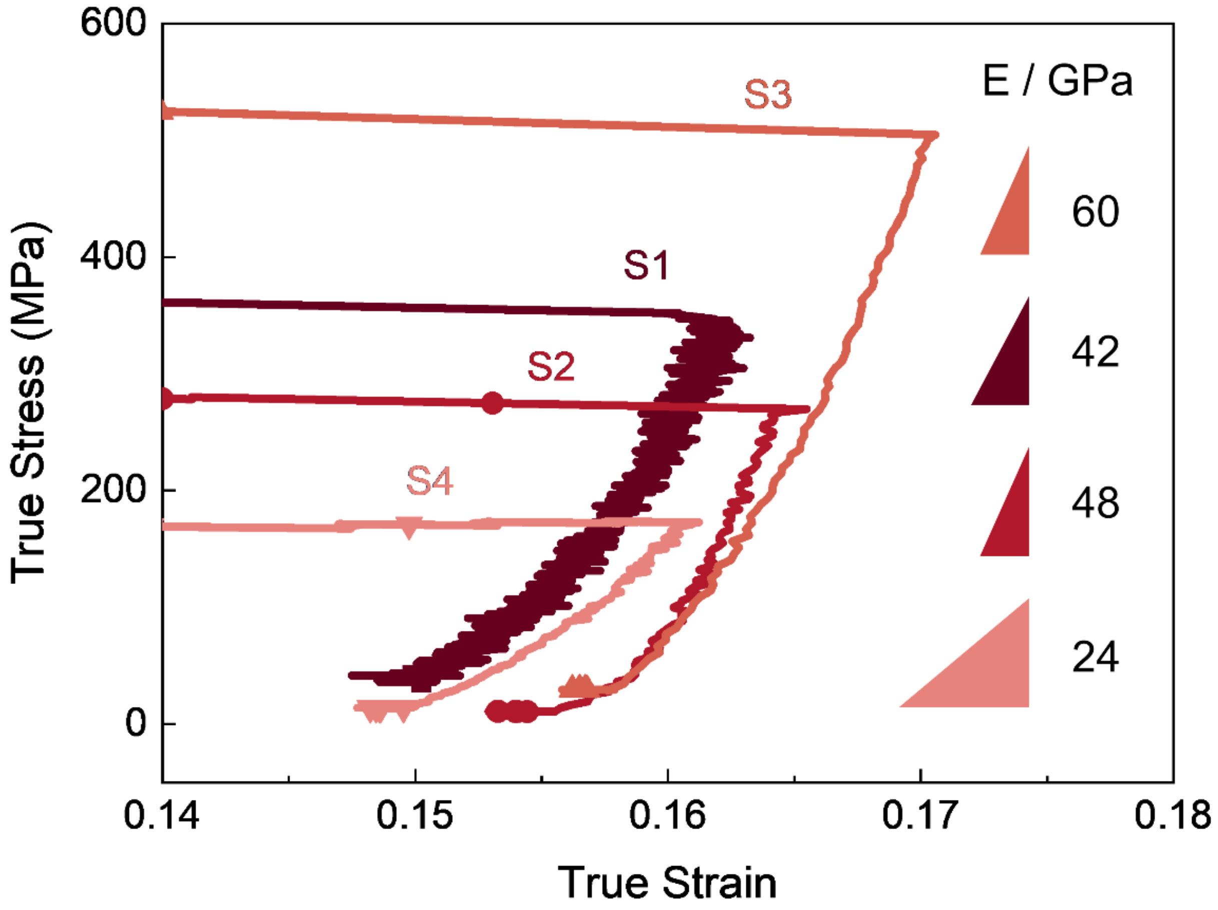

Supplementary Fig. 9 | Estimation of Young's modulus in the micro-pillars based on the unloading curve. In spite that Young's modulus of the micro-pillars is lower than that of the bulk sample via nanoindentation test, one could still infer that the micro-pillar which has higher Young's modulus possessed higher CZ content.

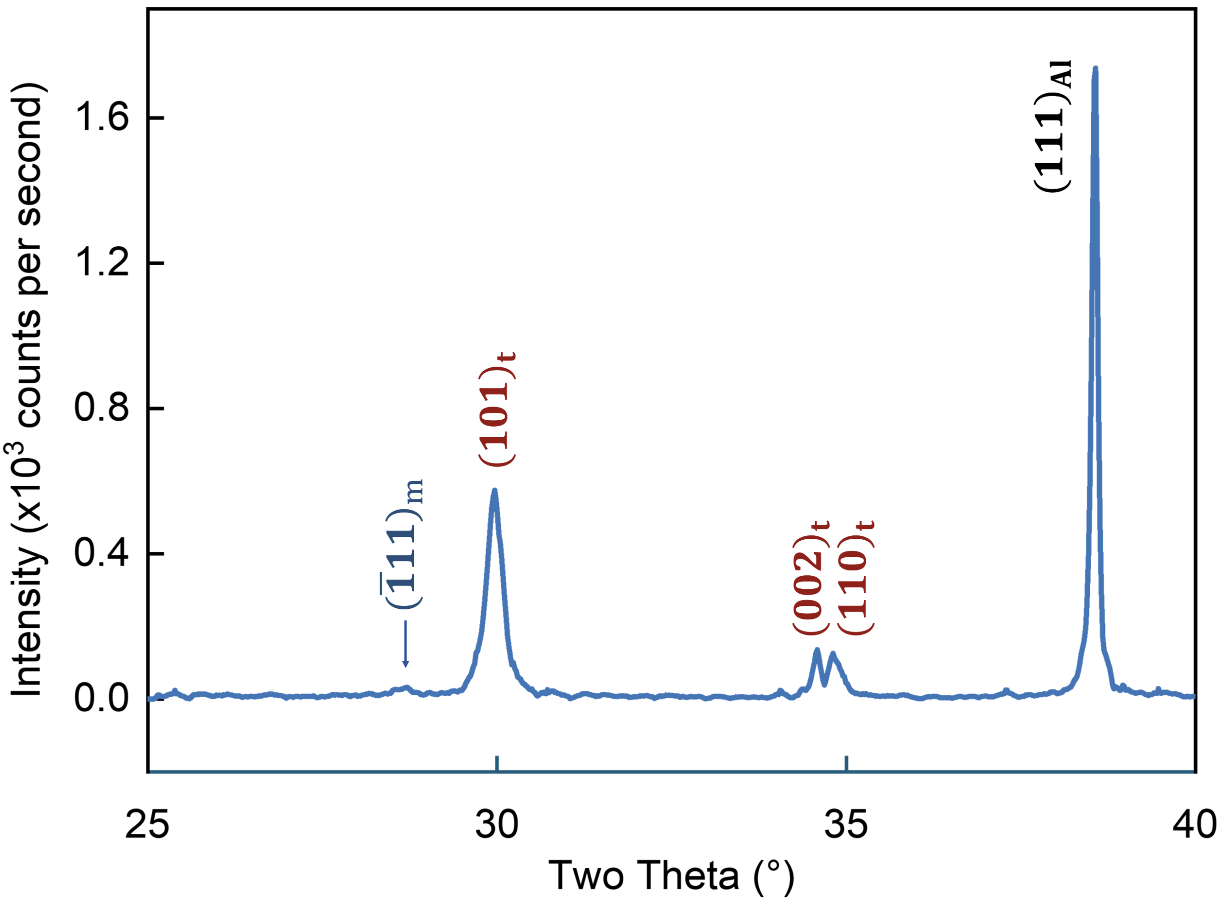

Supplementary Fig. 10 | XRD spectrums of bulk CZ/Al after uniaxial compression. The phase changes were slightly detected in the compressed bulk CZ/Al composite, as indicated by the relatively small peak at ~28° in the XRD pattern. Caution has to be taken that the conventional XRD measurement on bulk samples can only probe a thin depth (up to ~10 μm) into the specimen, and cannot provide a reliable estimate on the total amount of transformed CZ in the composite.

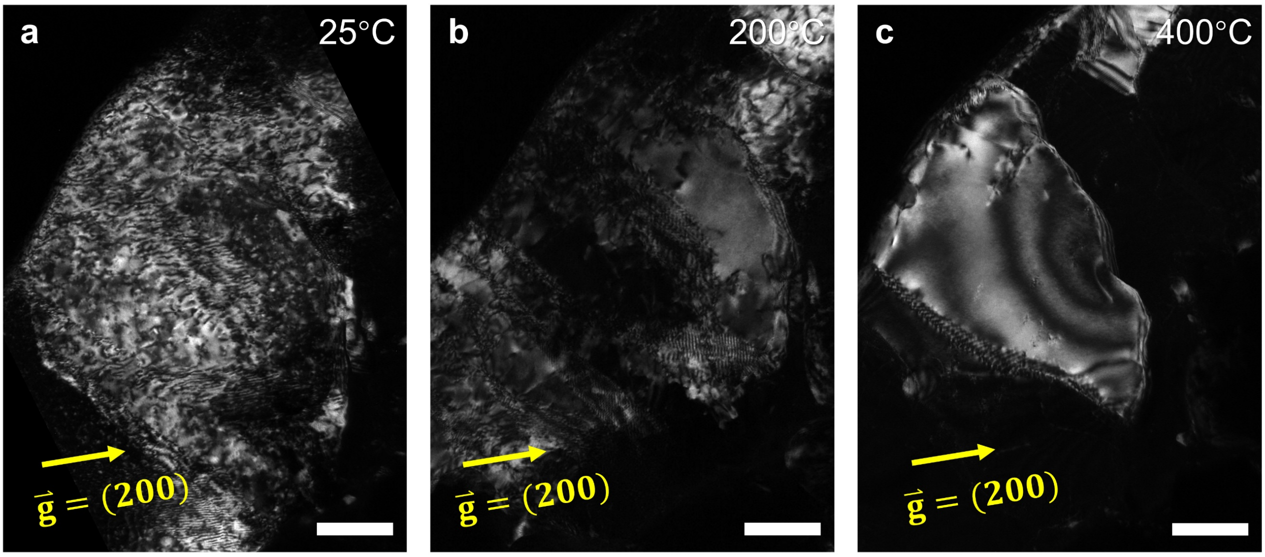

Supplementary Fig. 11 | Evolution on the dislocation during the thermally-induced reverse martensitic transformation in post-compression micro-pillar S1. Typical dark field (DF) TEM images of the dislocation structure in the Al region near the CZ particles with the state of **a** post-compression (25°C), and after annealing at **b** 200°C and **c** 400°C. All the specimens were taken under the dual-beam condition from the [011] zone axis and the (200) diffraction vector of Al. With the temperature elevating, dislocations within the Al grain have gradually disappeared with the relaxation of matrix constraint, while no interfacial delamination or fracture across the matrix was found. The scale bars in **a-c** are all 200 nm.

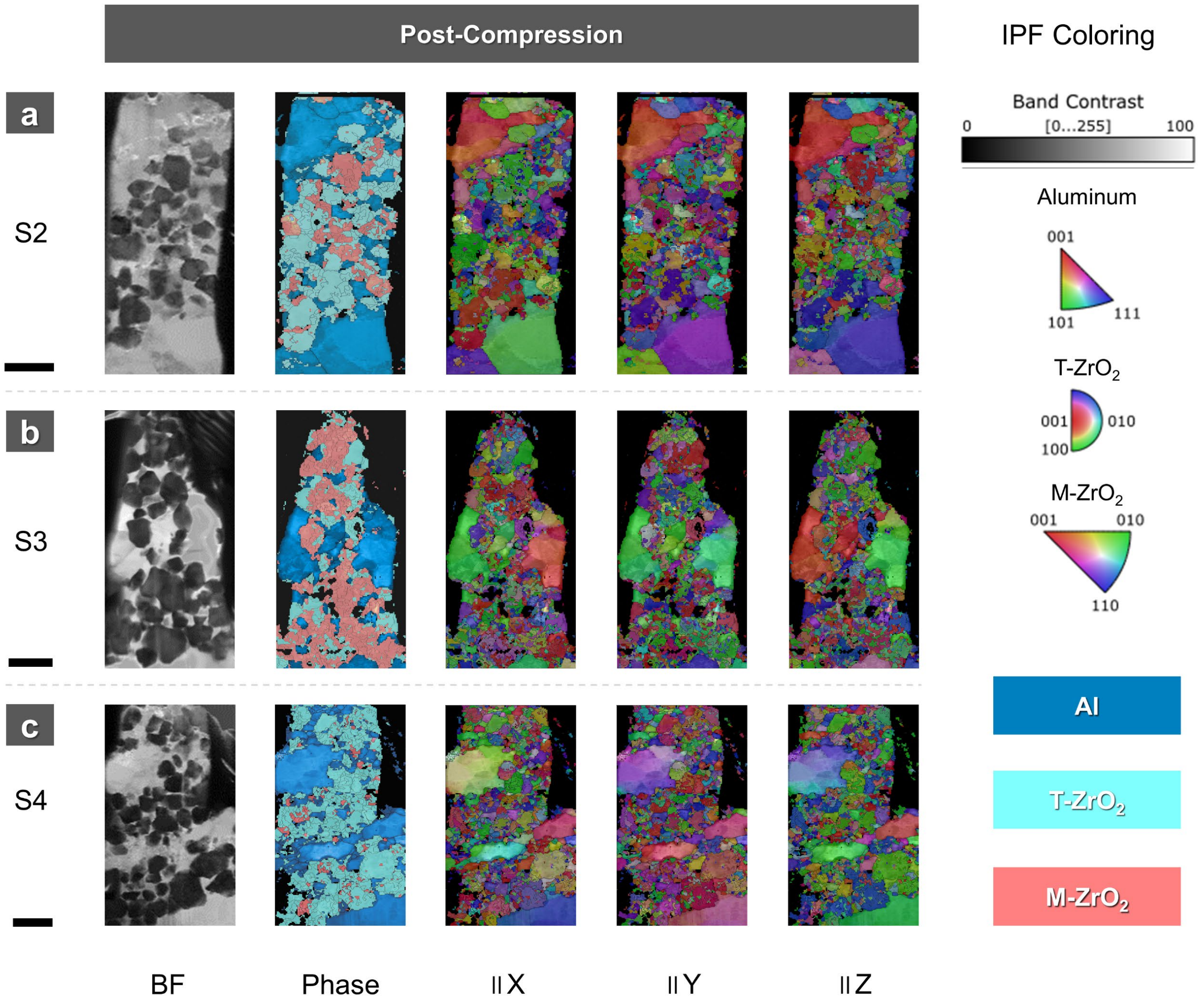

Supplementary Fig. 12 | Phase constitute and crystal orientation of the cross-section lamella of micro-pillars S2-S4 after compression. Precession electron diffraction (PED) on TEM cross-section lamella of post-compression micro-pillars **a** S2, **b** S3, and **c** S4. The bright field (BF) image, phase mapping, and inverse pore figures (IPF) at the x, y, and z axis were presented. Stress-induced martensitic transformation occurred in all three typical pillars. Tetragonal-T (light blue), monoclinic-M (pink), aluminum-Al (dark blue). The scale bars in **a**-**c** are all 1 μm.

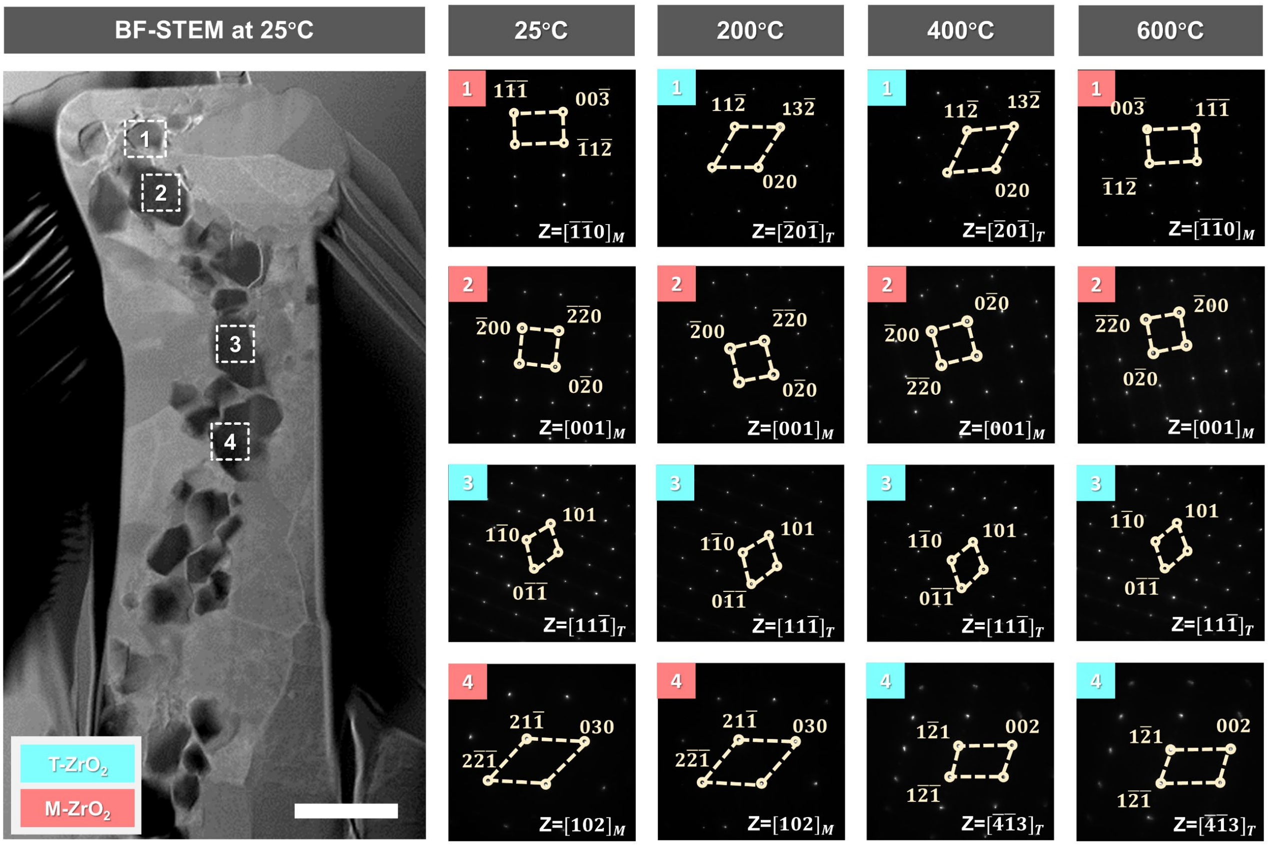

Supplementary Fig. 13 | Thermally-induced reverse martensitic transformation in micro-pillar S1. Selected area electron diffraction (SAED) patterns of #1-#4 CZ particles with the state of post-compression (25°C), annealing at 200°C (lower than $A_s = 304$°C), 400°C (between A_s and $A_f = 470$°C), 600°C (above A_f). The corresponding SAED patterns of #1-#4 particles presented are in line with the PED results in Fig. 5. After further annealing at 600°C, #1 particle switched to the monoclinic phase again, probably as a result of the more significant softening of Al and the subsequent relaxation on the geometrical constraint near the pillar top. Tetragonal-T (light blue), monoclinic-M (pink). The scale bar in BF-STEM image is 1 μm.

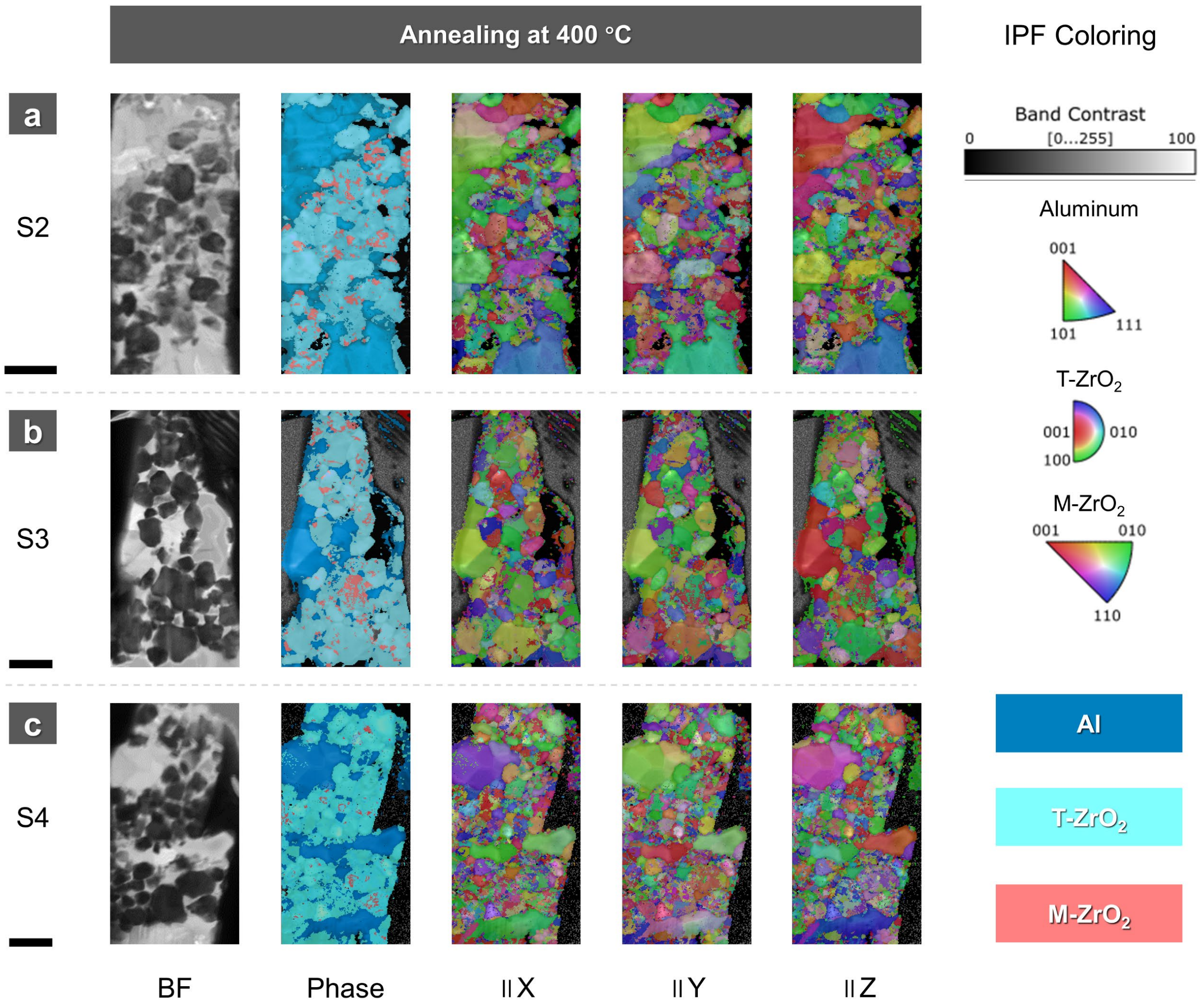

Supplementary Fig. 14 | Phase constitute and crystal orientation of the cross-section lamella of micro-pillars after annealing at 400°C. **a** S2, **b** S3, and **c** S4. The bright field (BF) image, phase mapping, and inverse pore figures (IPF) at the x, y, and z axis are presented. The transformational behavior of the three micro-pillars S2, S3, and S4 after 400°C annealing agreed with that of S1 (Supplementary Fig. 13). More than half of the transformed particles (50%, 79%, and 62%, respectively) were converted back to the tetragonal phase, respectively. Tetragonal-T (light blue), monoclinic-M (pink), aluminum-Al (dark blue). The scale bars in **a-c** are all 1 μm.

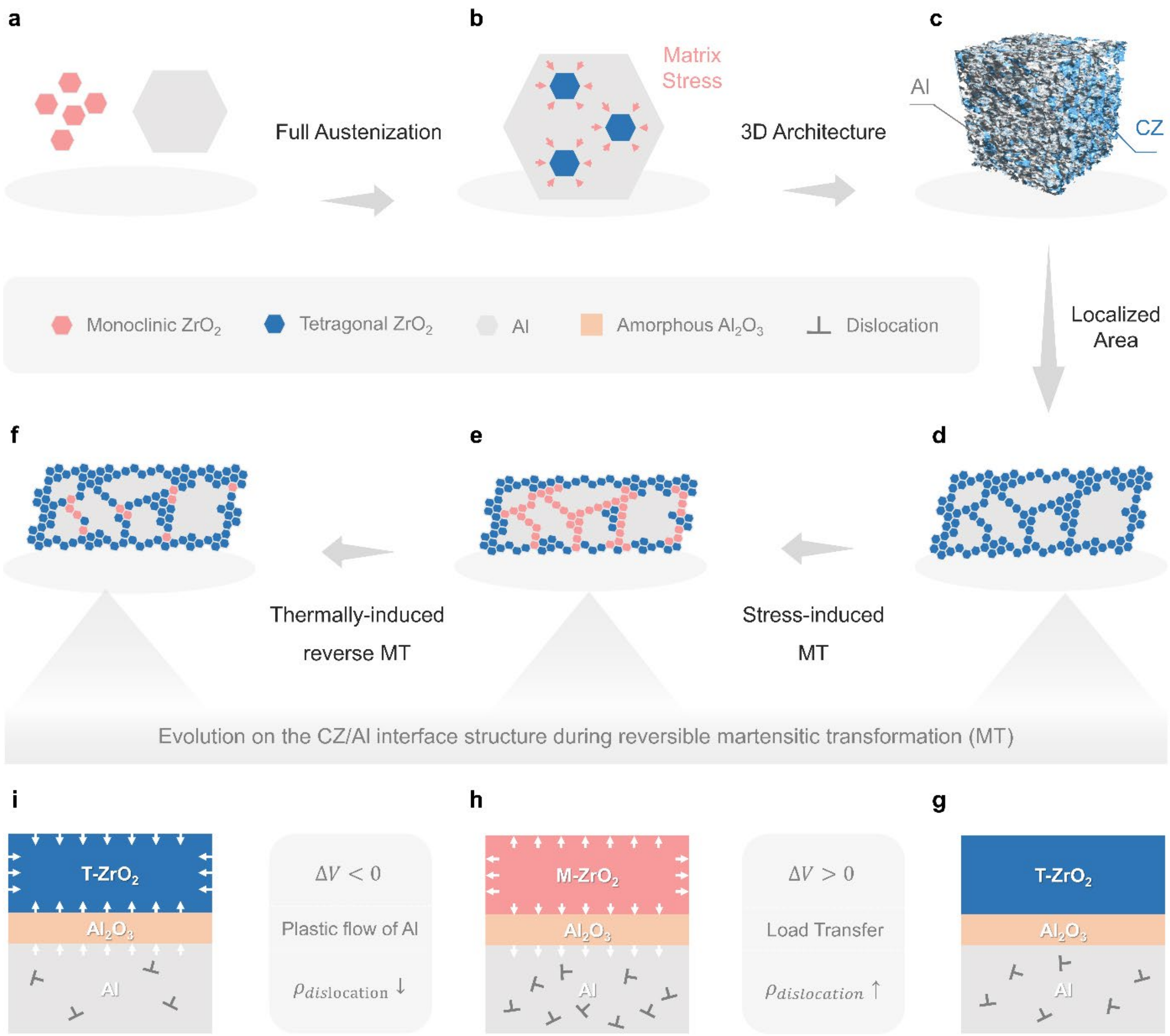

Supplementary Fig. 15 | Schematic mechanism of full austenization and reversible martensitic transformation in shape memory ceramics constraint in Al. **a** Monoclinic zirconia and Al. **b** Tetragonal zirconia stabilized by the matrix constraint. **c** 3D architecture of as-fabricated composites. **d** A representative local region with densely populated zirconia particles. **e** Stress-induced martensitic transformation (MT) and **f** thermally induced reverse martensitic transformation. The interfacial structure corresponding to **d-f** are magnified in **g-i**, where the white arrows correspond to the direction of volume expansion/contraction. The textboxes suggest the structure and property evolution during the thermo-mechanical treatments, i.e., the reversible phase transformations. Note: Tetragonal-T (blue), monoclinic-M (pink), aluminum-Al (grey).

Supplementary Tables

Supplementary Table 1 | Summary of the mechanical properties of as-fabricated CZ/Al and pure Al in the bulk form.

	**Density /g·cm^{-3}**	**Indentation Modulus /GPa**	**Indentation Hardness /GPa**	**HV**
**CZ/Al**	3.22 (100%)	89.2 ± 2.2	0.864 ± 0.038	82.5 ± 0.5
**Al**	2.70 (100%)	77.1 ± 0.9	0.603 ± 0.012	47.6 ± 0.4

Supplementary Table 2 | Full-width at half-maximum (FWHM), diffraction peak angle, and dislocation density corresponding to aluminum (111) peak in CZ/Al composites under multiple thermal cycles.

	FWHM/°	Diffraction Peak Angle/°	Dislocation density/10^{12} m^{-2}
**C0**	0.11	38.42	3.82
**C1**	0.18	38.58	5.95
**C5**	0.20	38.71	6.67
**C10**	0.26	38.82	8.63

Supplementary Table 3 | Summary of CZ particle content and distribution in the pillar, and the mechanical and transformational behaviors of micro-pillars S1 to S4. The actual CZ content in each pillar was estimated from the corresponding cross-section STEM images. The estimated CZ volume fractions in the micro-pillars were likely to be their upper limit, as the particles' dimension in the out-of-plane direction is smaller than the pillar's dimension.

	**Micro-pillar**	**S1**	**S2**	**S3**	**S4**
**Nature of CZ particles**	CZ Distribution	Arc	interconnected clustering	Double arc	Laminated
	Force-chain?	Yes	Yes	Yes	No
	CZ Content/vol.%	30	67	70	55
**Mechanical Behavior**	Compressive Strength/MPa *(5% flow stress)*	391	282	545	180
	Energy absorption/ $MJ\cdot m^{-3}$	54.0	37.5	74.8	24.8
	Deformation mode	Load transfer through CZ chains local extrusion of Al			Al shear
**Transformational Behavior**	Mechanically transformed fraction *(Mono. ratio)*	61%	30%	68%	13%
	Mono. ratio after 400°C	31%	15%	14%	5%
	Thermally transformed fraction *(Tetra. ratio)*	30%	15%	54%	8%
	Reversible transformation?	Yes	Yes	Yes	Yes

Supplementary References

1. Williamson GK, Smallman RE. III. Dislocation densities in some annealed and cold-worked metals from measurements on the X-ray debye-scherrer spectrum. *The Philosophical Magazine: A Journal of Theoretical Experimental and Applied Physics* **1**, 34-46 (1956).
2. Erb DJ, Rauch HA, Knight KP, Yu HZ. Viewpoint: Tuning the Martensitic Transformation Mode in Shape Memory Ceramics via Mesostructure and Microstructure Design. *Shape Memory and Superelasticity*, (2023).
